# Association of* NEFL* Gene Polymorphisms with Wilms' Tumor Susceptibility in Chinese Children

**DOI:** 10.1155/2019/3518149

**Published:** 2019-04-01

**Authors:** Wei Jia, Jinhong Zhu, Wen Fu, Shibo Zhu, Fuming Deng, Huimin Xia, Guo-Chang Liu, Jing He

**Affiliations:** ^1^Department of Pediatric Urology, Guangzhou Women and Children's Medical Center, Guangzhou Medical University, Guangzhou 510623, Guangdong, China; ^2^Department of Clinical Laboratory, Molecular Epidemiology Laboratory, Harbin Medical University Cancer Hospital, Harbin 150040, Heilongjiang, China; ^3^Department of Pediatric Surgery, Guangzhou Institute of Pediatrics, Guangzhou Women and Children's Medical Center, Guangzhou Medical University, Guangzhou 510623, Guangdong, China

## Abstract

Wilms' tumor is renal tumor of childhood, characterized by the appearance of embryonic renal tissue and other kidney malformations. The genetic etiology of sporadic Wilms' tumor remains largely unknown. Neurofilament light (NEFL) is a tumor suppressor. We evaluated the association between three* NEFL* gene polymorphisms (rs11994014 G>A, rs2979704 T>C and rs1059111 A>T) and Wilms' tumor susceptibility in a Chinese population consisting of 145 cases and 531 controls. In the single locus analysis, rs2979704 CC variant genotype was associated with a decreased risk of Wilms' tumor [CC vs. TT: adjusted odds ratio (OR)=0.48, 95% confidence interval (CI)=0.24-0.94; CC vs. TT+CT: adjusted OR=0.51, 95% CI=0.27-0.97]. We also observed that carriers of the three protective genotypes had significantly decreased risk of Wilms' tumor when compared to those with 0-2 protective genotypes (adjusted OR=0.49, 95% CI=0.25-0.95). The association between rs11994014 G>A or rs1059111 A>T polymorphisms and Wilms' tumor susceptibility did not reach statistical significance. No significant association was detected in the stratified analyses. Our findings suggested that the* NEFL* rs2979704 T>C polymorphism may be associated with Wilms' tumor susceptibility in the Chinese population.

## 1. Introduction

Wilms' tumor (nephroblastoma), one of the most common pediatric solid tumors, is an embryonal tumor of kidney. It approximately affects one in 10000 children, with 90% of cases occurring before the age of 7 years [1]. The clinical outcome of Wilms' tumor is closely related to its histological differentiation and malignancy grade. With a combination of surgery, chemotherapy, and radiation therapy, this disease reaches a cure rate greater than 85%, to date. However, prognosis of patients with a poor degree of tumor differentiation remains unsatisfying, such as the high-risk group [2]. Wilms' tumors develop from pluripotent embryonic renal precursor cells, featured by the copresence of “triphasic” histology, comprising blastemal, epithelial, and stromal components. During the development of malignancy, metanephric mesenchyma fails to undergo mesenchymal-to-epithelial transition to form nephrons of the kidney [3]. Genetic abnormalities identified in Wilms' tumor, especially tumor suppressor genes, are believed to be closely connected to kidney organogenesis [4]. There is strong evidence that genetic factors may contribute to the development of Wilms' tumor, but only approximately 5% of cases harbor defined gene mutations. Genetic basis underlying the majority of Wilms' tumor remains largely unknown [5]. A genome-wide association study (GWAS) by Turnbull et al. [6] only identified one Wilms' tumor susceptibility gene,* DDX1* in 757 cases and 1,879 controls of European ancestry. Authors also strongly suggested that multiple loci conferring equivalent or weaker susceptibility are likely to exist. Thus, lots of loci have been identified for neuroblastoma [7–12], another embryonic tumor. These loci may be identified through follow-up analysis of additional single nucleotide polymorphisms (SNPs) showing evidence of association in this study and/or through further GWAS or candidate gene approaches or fine mapping, such as polymorphisms in* CDKN1B* and* BARD1* genes were identified to be associated with neuroblastoma susceptibility [13, 14]. Despite that, these risk variants only explain heritability for a small proportion of embryonic tumor and additional predisposing variants to remain to be clarified [1, 5].

The human* neurofilament light *(*NEFL*) gene encodes the light subunit of neurofilaments, which functionally maintain neuronal caliber and regulate intracellular transport to axons and dendrites [15].* NEFL* gene is located on chromosome 8p21, a region enriched with tumor suppressor genes. In addition to its influence on the nervous system,* NEFL* gene has been also involved in the spontaneous tumorigenesis in human [16–18]. Three polymorphisms (rs11994014 G>A, rs2979704 T>C and rs1059111 A>T) within the* NEFL* gene has been demonstrated to influence neuroblastoma susceptibility and disease progression [19]. Given the important tumor-suppressing role of the* NEFL* gene, we investigated the potential association between these three* NEFL* polymorphisms and Wilms' tumor susceptibility in a Chinese population including 145 cases and 531 controls.

## 2. Materials and Methods

### 2.1. Study Participants and Ethical Statement

The study was approved by the Institutional Review Board of Guangzhou Women and Children's Medical Center. We enrolled a total of 145 newly diagnosed and histopathologically confirmed Wilms' tumor children cases from the Guangzhou Women and Children's Medical Center between March 2001 and June 2016 [20–24]. In addition, 531 cancer-free children were selected as the age- and gender-matched controls from those receiving routine physical examination during the same period [25–28]. Information of cases and controls was listed in Supplemental Table 1. Both the cases and the controls were unassociated ethnic Chinese Han individuals. Demographic factors and medical histories were obtained using a structured questionnaire and written informed consent was obtained from parents of each participant.

### 2.2. SNPs Selection and Genotyping

Three polymorphisms (rs11994014 G>A, rs2979704 T>C, rs1059111 A>T) within the* NEFL* gene were selected based on previous publications [19, 29]. Potential functions of these SNPs were predicted by SNPinfo online tool (https://snpinfo.niehs.nih.gov/snpinfo/snpfunc.html), and results were listed in Supplemental Table 2. As shown in Supplemental Figure 1, using data from LDlink online software (https://ldlink.nci.nih.gov/?tab=ldmatrix), there existed significant linkage disequilibrium (R^2^ > 0.8) between these three SNPs (R^2^=1.00 between rs11994014 and rs2979704; R^2^=0.99 between rs11994014 and rs1059111; R^2^=0.99 between rs2979704 and rs1059111). Genomic DNA was mainly isolated from the peripheral blood samples with a TIANamp Blood DNA Kit (TianGen Biotech, Beijing, China). A 7900 Sequence Detection System (Applied Biosystem, Foster City, CA) and Taqman real-time PCR were used to genotype the* NEFL *SNPs, as described previously [30–32]. Four duplicate positive controls and four negative controls without DNA template were loaded in each 96-well plate. To verify genotyping results, genotyping was repeated on 10% of randomly selected samples, and the results were 100% concordant [33].

### 2.3. Statistical Analysis

The chi-square test was used to evaluate the differences in the frequency distributions of the demographics and genotypes between cases and controls. The unconditional multivariate logistic regression was applied. Odds ratios (ORs) and 95% confidence intervals (CIs) were used to assess the strength of associations between the three polymorphisms and Wilms' tumor susceptibility. Stratified analysis was performed by age, gender, and clinical stages. All statistical tests were two-sided.* P* < 0.05 was considered statistically significant. All statistical analyses were performed using SAS software (version 9.1; SAS Institute, Cary, NC) [34–36].

## 3. Results

### 3.1. Associations of* NEFL* Gene Polymorphisms with Wilms' Tumor Susceptibility

Detailed information on cases and controls were reported in the previous studies [20–24]. Totally, 144 cases and 531 controls were successfully genotyped. The mean age was 26.17±21.48 months for the Wilms' tumor cases and 29.73±24.86 months for controls. According to the classification of Wilms' tumor defined by NWTS-5 criteria, the cases were classified into clinical stages I, II, III, and IV [37]. The genotype distributions for the three SNPs and their associations with Wilms' tumor susceptibility are summarized in Table 1. All frequency distributions of all of the SNPs were consistent with Hardy-Weinberg equilibrium (*P*=0.258 for rs11994014 G>A,* P*=0.245 for rs2979704 T>C, and* P*=0.275 for rs1059111 A>T polymorphism) in control subjects. In the single locus analysis, rs2979704 CC variant genotype was associated with a decreased risk of Wilms' tumor (adjusted OR=0.48; 95% CI=0.24-0.94,* P*=0.031), when compared with TT genotype. The association remained significant under the recessive model (adjusted OR=0.51; 95% CI=0.27-0.97,* P*=0.041). No significant associations were observed for the other two SNPs. Although association was not significant, these two polymorphism appeared to be protective against Wilms' tumor as indicated by ORs <1. Therefore, we also defined rs11994014 AA and rs1059111 TT as protective genotypes.

Moreover, when the subjects were divided into two groups (0-2 verse 3 risk genotypes), we found the subjects with 3 protective genotypes had a significantly decreased Wilms' tumor risk in comparison with those with 0-2 protective genotypes (adjusted OR=0.49, 95% CI=0.25-0.95,* P*=0.035).

### 3.2. Stratification Analysis of* NEFL* Gene Polymorphisms with Wilms' Tumor Susceptibility

In stratified analysis by age, gender, and clinical stages, we further assessed the potential association between* NEFL* gene rs2979704 T>C, rs11994014 G>A polymorphisms and combined protective genotypes with Wilms' tumor susceptibility (Table 2). However, no significant association was identified.

## 4. Discussion

In this hospital-based case-control study, we systematically evaluated the associations between three* NEFL* gene polymorphisms and Wilms' tumor susceptibility in 145 patients and 531 controls. We found that carriers of rs2979704 CC genotype and the three protective genotypes had significantly decreased risk of Wilms' tumor when compared to respective reference groups. Moreover, the association between rs11994014 G>A or rs1059111 A>T polymorphisms and Wilms' tumor susceptibility did not reach statistical significance. Similarly, no significant association was discovered in the stratified analyses. To the best of our knowledge, this is the first study to demonstrate such associations in a Chinese population.

The protein product of* NEFL* gene is the neurofilament light chain. Mutations in the* NEFL* cause the most severe Charcot-Marie-Tooth disease types 1F and 2E disorders of the peripheral nervous system. These diseases are characterized by distinct neuropathies [37]. Recently, the NEFL protein is implicated in the pathophysiology of several tumors as a potential tumor suppressor. For instance, homozygous deletions of chromosomal region 8p21, where* NEFL* is located, have been detected in prostate cancer and squamous cell carcinoma of the head and neck [38, 39]. Moreover, NEFL was also found to participate in the development and chemical resistance of a variety of common human cancers, including breast cancer and colorectal cancer [18, 40, 41]. In glioma, overexpression of NEFL significantly suppressed the proliferation and invasion of cancer cells and enhanced the chemosensitivity of glioblastoma cells to temozolomide [42].

Additionally, some studies have demonstrated the association between* NEFL* gene polymorphisms and cancer risk susceptibility. Kerangueven et al. [43] has reported that several loci on the short arm of chromosome within the* NEFL* gene are associated with breast carcinoma susceptibility by loss of heterozygosity and linkage analysis. Recently, Capasso et al. [19] replicated the GWAS to examine the cumulative moderate effect of* NEFL* genetic variations on neuroblastoma susceptibility in an Italian population (370 cases and 809 controls). Turnbull et al. [6] using the GWAS approach only identified* DDX1* as a Wilms' tumor susceptibility gene in 757 Wilms' tumor cases and 1,879 controls of European ancestry. Although undetectable by GWASs, low-frequency coding variants could also be functional and confer susceptibility to diseases.

Several limitations should be acknowledged in our study when interpreting the findings. First, the sample size in this study, especially the Wilms' tumor cases, is relatively small, which may affect the power in statistical analysis. Multicenter studies with large sample size are warranted to assess the association. Second, more potentially functional polymorphisms within the* NEFL* gene should be evaluated as predicted by SNPinfo online software. Finally, because of the retrospective study design, we could not collect and analyze some important factors, such as parental environmental exposures.

## 5. Conclusions

In summary, our present data indicated the* NEFL* gene polymorphisms may associate with Wilms' tumor susceptibility in the Chinese population. In the future, prospective studies with larger sample size and more SNPs should be performed to confirm our findings.

## Figures and Tables

**Table 1:** Associations between *NEFL* gene polymorphisms and Wilms' tumor susceptibility.

Genotype	Cases	Controls	*P* ^a^	Crude OR	*P*	Adjusted OR^b^	*P* ^b^
	(N=144)	(N=531)		(95% CI)		(95% CI)	
rs11994014 G>A (HWE=0.258)							
GG	59 (40.97)	186 (35.03)		1.00		1.00	
AG	72 (50.00)	267 (50.28)		0.85 (0.58-1.26)	0.417	0.85 (0.57-1.26)	0.422
AA	13 (9.03)	78 (14.69)		0.53 (0.27-1.01)	0.055	0.52 (0.27-1.01)	0.052
Additive			0.150	0.77 (0.58-1.02)	0.065	0.76 (0.58-1.02)	0.063
Dominant	85 (59.03)	345 (64.97)	0.188	0.78 (0.53-1.13)	0.189	0.78 (0.53-1.14)	0.191
Recessive	131 (90.97)	453 (85.31)	0.078	0.58 (0.31-1.07)	0.081	0.57 (0.31-1.06)	0.076
rs2979704 T>C (HWE=0.245)							
TT	58 (40.28)	184 (34.65)		1.00		1.00	
CT	74 (51.39)	268 (50.47)		0.88 (0.59-1.30)	0.508	0.87 (0.59-1.29)	0.499
CC	12 (8.33)	79 (14.88)		**0.48 (0.25-0.95)**	**0.034**	**0.48 (0.24-0.94)**	**0.031**
Additive			0.099	0.76 (0.57-1.00)	0.052	0.75 (0.57-0.999)	0.049
Dominant	86 (59.72)	347 (65.35)	0.212	0.79 (0.54-1.15)	0.212	0.78 (0.54-1.15)	0.207
Recessive	132 (91.67)	452 (85.12)	0.041	**0.52 (0.28-0.98)**	**0.045**	**0.51 (0.27-0.97)**	**0.041**
rs1059111 A>T (HWE=0.275)							
AA	58 (40.28)	185 (34.84)		1.00		1.00	
AT	70 (48.61)	267 (50.28)		0.84 (0.56-1.24)	0.375	0.84 (0.56-1.25)	0.380
TT	16 (11.11)	79 (14.88)		0.65 (0.35-1.19)	0.163	0.63 (0.34-1.18)	0.148
Additive			0.344	0.81 (0.62-1.08)	0.146	0.81 (0.61-1.07)	0.136
Dominant	86 (59.72)	346 (65.16)	0.228	0.79 (0.54-1.16)	0.228	0.79 (0.54-1.16)	0.226
Recessive	128 (88.89)	452 (85.12)	0.249	0.72 (0.40-1.27)	0.251	0.70 (0.40-1.25)	0.228
Combined effect of protective genotypes							
0-2	133 (92.36)	455 (85.69)		1.00			
3	11 (7.64)	76 (14.31)	0.034	**0.50 (0.26-0.96)**	**0.037**	**0.49 (0.25-0.95)**	**0.035**

OR, odds ratio; CI, confidence interval; HWE, Hardy-Weinberg equilibrium.

^a^ *χ* ^2^ test for genotype distributions between Wilms' tumor patients and cancer-free controls.

^b^Adjusted for age and gender.

^c^Protective genotypes were rs11994014 AA, rs2979704 CC, and rs1059111 TT.

**Table 2:** Stratification analysis of protective genotypes and Wilms' tumor susceptibility.

Variables	rs11994014		Adjusted OR^a^	*P* ^a^	rs2979704		Adjusted OR^a^	*P* ^a^	Combined genotypes		Adjusted OR^a^	*P* ^a^
	(cases/controls)				(cases/controls)				(cases/controls)			
	GG+AG	AA	(95% CI)		TT+CT	CC	(95% CI)		0-2	3	(95% CI)	
Age, month												
≤18	59/199	6/34	0.59 (0.24-1.47)	0.256	60/198	5/35	0.47 (0.18-1.25)	0.130	61/199	4/34	0.38 (0.13-1.12)	0.079
>18	72/254	7/44	0.58 (0.25-1.34)	0.201	72/254	7/44	0.58 (0.25-1.34)	0.201	72/256	7/42	0.61 (0.26-1.42)	0.253
Gender												
Females	60/206	4/27	0.50 (0.17-1.49)	0.213	59/206	5/27	0.64 (0.23-1.73)	0.374	60/207	4/26	0.52 (0.17-1.55)	0.240
Males	71/247	9/51	0.61 (0.29-1.31)	0.207	73/246	7/52	0.45 (0.20-1.04)	0.063	73/248	7/50	0.48 (0.21-1.10)	0.083
Clinical stages												
I+II	48/453	5/78	0.61 (0.23-1.58)	0.307	49/452	4/79	0.47 (0.16-1.33)	0.154	49/455	4/76	0.48 (0.17-1.39)	0.178
III+IV	76/453	6/78	0.45 (0.19-1.07)	0.072	76/452	6/79	0.44 (0.19-1.06)	0.066	76/455	6/76	0.47 (0.20-1.11)	0.084

OR, odds ratio; CI, confidence interval.

^a^Adjusted for age and gender, omitting the corresponding stratification factor.

## Data Availability

The data used to support the findings of this study are available from the corresponding author upon request.

## Abbreviations

GWAS: Genome-wide association study
SNP: Single nucleotide polymorphism
*NEFL*: * Neurofilament light*
OR: Odds ratio
CI: Confidence interval.

## Supplementary Materials

Supplementary Materials Supplemental Figure 1: linkage disequilibrium analysis for the three selected polymorphisms in* NEFL* gene in Han Chinese population consisted of CHB (Han Chinese in Beijing, China) and CHS (Southern Han Chinese) subjects using data from LDlink online software (https://ldlink.nci.nih.gov/?tab=ldmatrix). Supplemental Table 1: frequency distribution of selected variables for Wilms' tumor cases and cancer-free controls. Supplemental Table 2: potential functions of the three selected* NEFL* polymorphisms as predicted by SNPinfo online software (https://snpinfo.niehs.nih.gov/snpinfo/snpfunc.html).
