# Supplementary material for: Association of NEFL Gene Polymorphisms with Wilms' Tumor Susceptibility in Chinese Children
Source: J Oncol. 2019 Apr 1;2019:3518149. doi: 10.1155/2019/3518149 (PMC6463584; doi:10.1155/2019/3518149)
Supplement: Supplementary Materials — Supplemental Figure 1: linkage disequilibrium analysis for the three selected polymorphisms in NEFL gene in Han Chinese population consisted of CHB (Han Chinese in Beijing, China) and CHS (Southern Han Chinese) subjects using data from LDlink online software (https://ldlink.nci.nih.gov/?tab=ldmatrix). Supplemental Table 1: frequency distribution of selected variables for Wilms' tumor cases and cancer-free controls. Supplemental Table 2: potential functions of the three selected NEFL polymorphisms as predicted by SNPinfo online software (https://snpinfo.niehs.nih.gov/snpinfo/snpfunc.html). [file 3518149.f1.pdf]

Supplemental Table 1: Frequency distribution of selected variables for Wilms' tumor cases and cancer-free controls.

Variables	Cases (n=145)		Controls (n=531)		P^{a}
	No.	%	No.	%	
Age range, month	1-132		0.07-156		0.725
Mean ± SD	26.17 ± 21.48		29.73 ± 24.86		
≤18	66	45.52	233	43.88	
>18	79	54.48	298	56.12	
Gender					0.956
Female	64	44.14	233	43.88	
Male	81	55.86	298	56.12	
Clinical stages					
I	4	2.76			
II	49	33.79			
III	50	34.48			
IV	33	22.76			
NA	9	6.21			

SD, standard deviation; NA, not available.

[a] Two-sided χ^2 test for distributions between Wilms' tumor cases and cancer-free controls.

Supplemental Table 2: Potential functions of the three selected *NEFL* polymorphisms as predicted by SNPinfo online software (https://snpinfo.niehs.nih.gov/snpinfo/snpfunc.html).

rs	Chromosome	Allele	TFBS	miRNA (miRanda)	Allele	Asian	CHB
rs11994014	8	A/G	--	--	G	0.649	0.613
rs2979704	8	C/T	Y	Y	T	0.605	0.614
rs1059111	8	T/A	Y	Y	A	0.606	0.567

TFBS, transcription factor binding sites; CHB, Han Chinese in Beijing, China.

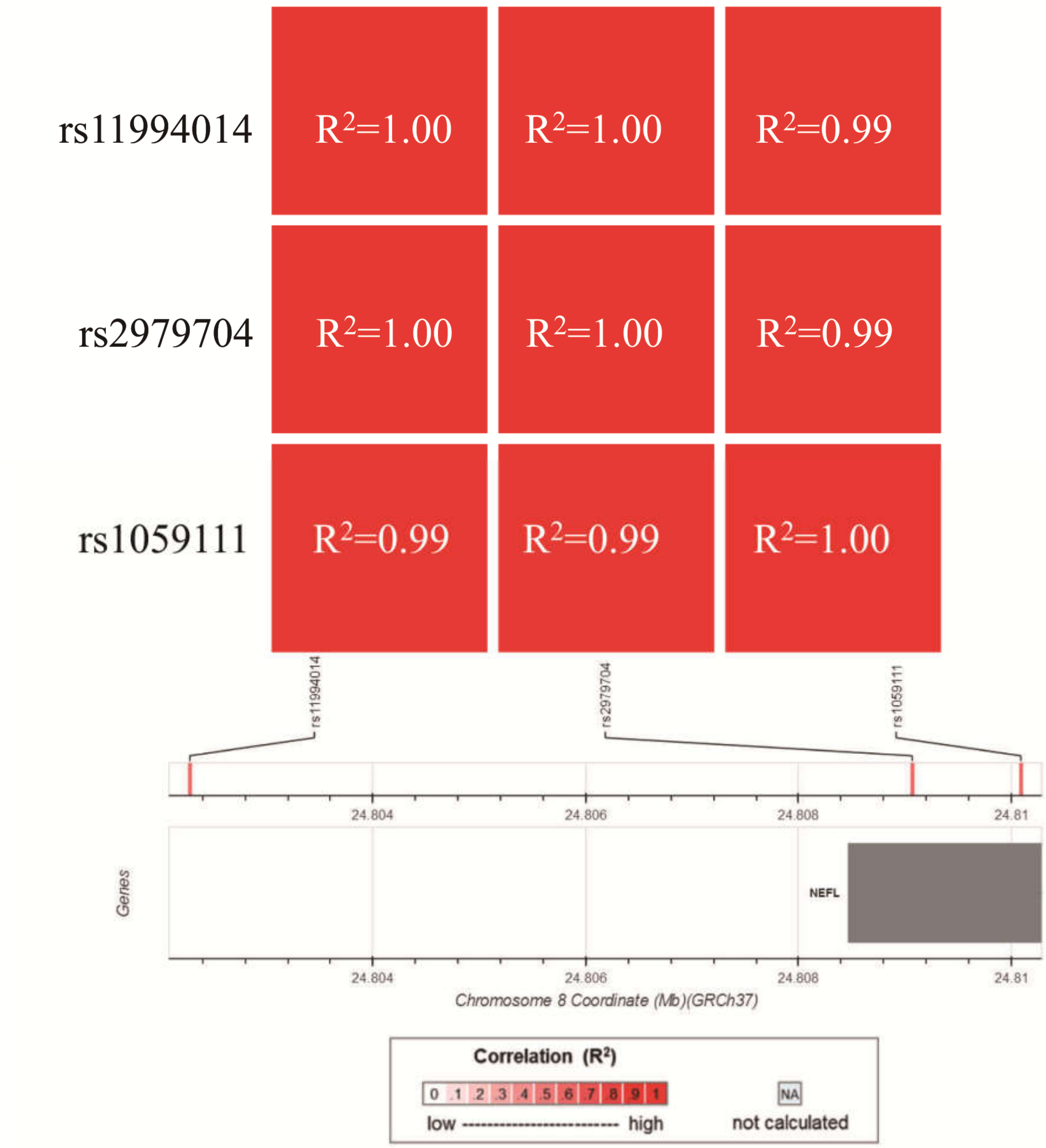

Supplemental Figure 1: Linkage disequilibrium analysis for the three selected polymorphisms in *NEFL* gene in Han Chinese population consisted of CHB (Han Chinese in Beijing, China) and CHS (Southern Han Chinese) subjects using data from LDlink online software (https://ldlink.nci.nih.gov/?tab=ldmatrix).
